# Quantitative analysis of the obturation of oval-shaped canals using thermoplastic techniques

**DOI:** 10.4317/jced.60491

**Published:** 2023-04-01

**Authors:** Nicolás Collado-Castellanos, Alba Aspas-García, Alberto Albero-Monteagudo, Alberto Manzano-Saiz, Pedro Micó-Muñoz

**Affiliations:** 1 Associate professor. Faculty of Health Sciences. Department of Dentistry. European University of Valencia. Spain; 2 Endodontic and restorative dentistry Associate Professor, Universidad Europea de Valencia, España; 3 Endodontic and restorative dentistry Titular Professor, Universidad Europea de Valencia, España

## Abstract

**Background:**

One of the aim of root canal treatment is filling the root canals in a 3D way. It is not always possible to achieve due to the existence of anatomical variations. The obturation of oval canal usually provides great difficulties. Its complex anatomy can cause obstacles in instrumentation, irrigation and obturation. Aim: The main aim of this study is to quantify the area filled by gutta-percha, the area filled by cement and the area of voids present in oval canals, treated by thermoplastic filling techniques in comparison with the lateral condensation technique and observe the adaptation of the gutta-percha to the anatomy of the oval root canals according to the obturation technique.

**Material and Methods:**

80 mandibular incisors were selected. The teeth were instrumented with Protaper Gold® and divided into 4 groups of 20. Group 1 was filled with Thermafill®; group 2 with GuttaCore®; group 3 with continuous wave vertical condensation and group 4 with the lateral condensation technique. Two horizontal sections were cut at 5mm and 7mm from the apex and they were set in putty silicone. The samples were analyzed with a Leica DMS 1000 digital microscope and processed with Leica Suite for Windows XP. The area of gutta-percha, cement and voids and the percentages, of each one, were calculated. A statistical analysis was performed using the T-Student, ANOVA tests and Kruskal-Wallis tests.

**Results:**

All thermoplastic techniques achieved a high percentage of obturation. The percentages of voids in group 4 were the highest. Statistically significant differences were found between thermoplastic techniques compared with lateral condensation.

**Conclusions:**

Thermoplastic techniques achieve better adaptation of the gutta-percha in the oval canals and low amount of cement and voids in the middle and coronal thirds respect to the lateral condensation group. Moreover, comparing termoplasthicized techniques among them, continuous wave vertical condensation got the lowest percentage of voids.

** Key words:**Warm gutta-percha, thermoplastic obturation, oval canals, oval-shaped canals.

## Introduction

One of the main objectives of endodontic treatment is the complete preparation of the root canal system and correct obturation to obtain a three-dimensional seal.

The anatomical configurations of root canals can vary: round, oval, flat or irregular. Each root morphology may require a different approach to cleaning, instrumentation and obturation (1).

Oval-shaped canals have a maximum diameter in the vestibulo-lingual (VL) direction up to twice the minimum diameter, mesio-distal diameter (MD). One variant of these canals is long oval-shaped canals, in which the maximum diameter (VL) is two to four times larger than the minimum diameter (MD) (2). A high prevalence of oval-shaped and long oval-shaped root canals (including in the apical area) has been reported. Long oval-shaped canals account for 25% of cases. This type of canal is found most frequently in mandibular incisors and maxillary premolars with a single canal, where the prevalence is as high as 50% (2). This complex anatomy can be considered one of the biggest challenge for adequate debridement in clinical practice.

The correct instrumentation of root canals is fundamental to achieving a good end result. However, due to anatomical variations, it is rarely fully achieved. Accordingly, oval-shaped canals can give rise to even more challenges (1). For example, applying the same shaping and cleaning protocol for round canals to oval-shaped canals may result in an incomplete preparation of the oval-shaped canal, leaving uninstrumented areas, mainly the vestibular and lingual extensions. Microtomography (micro-CT) studies reveal that between 5% and 80% of the root surface may remain uninstrumented, making subsequent obturation of the canal system difficult (3).

Some studies state that the shape of the root canal influences the sealing capacity in the short term. The obturation of oval-shaped canals is in itself a challenge for the practitioner (4). The obturation material is most effective when used in conjunction with a sealer, which can fill imperfections and increase the adaptation of the gutta-percha to the canal (5). Therefore, the quality of the apical seal is also affected by the amount of cement present. According to Wu MK *et al*., an optimal root obturation should maximise the volume of gutta-percha and minimise the amount of sealer (6), as sealer-filled areas are more vulnerable to dissolution, while gutta-percha is more stable (7). The appearance of new obturation materials and their constant evolution makes it necessary to study and compare the different techniques available on the market. To assess the sealing ability of gutta-percha and cement, several observation methods have been used. Observation methods include radiographs, bacterial filtration, cross-sections with subsequent microscopic observation and microtomography (8-11).

This study compares different obturation techniques in oval-shaped canals. The anatomical variations of these canals make treatment difficult, which is why it is both important and interesting to study and compare the different obturation techniques in order to find which is the most effective. This study has a very large sample size compared to most studies published in the current literature comparing four obturation techniques: Thermafil (TF), GuttaCore (GC), Continuous Wave Vertical Condensation (VC) and Lateral Condensation (LC).

The aim of this study was to quantify the area filled by gutta-percha, the area filled by sealer and the area of voids in oval-shaped canals treated by thermoplastic obturation techniques compared to the LC technique and observe the adaptation of the gutta-percha to the anatomy of the oval-shaped canals according to the obturation technique.

## Material and Methods

This study was approved by the ethics and research committee of the European University of Madrid under internal code number CIPI/18/177.

This *in-vitro* study was performed using lower incisors extracted for reasons unrelated to the study. 80 mandibular incisors with a single root canal were selected. The oval-shaped anatomy was confirmed with two radiographs in the LV and MD direction. Teeth with a single canal whose largest diameter was at least twice the smallest diameter were selected, and those with accessory canals were excluded. Both the instrumentation and the obturation were carried out by specialised dentist in endodontics.

After the access opening, a K-file No. 10 was used, and the working length (WL) was set at 1 mm from the apical foramen by visual observation. The glide path was made up to file No. 20, after using the permeability file No. 10, at each file pass. All the canals were prepared with Protaper Gold® (Dentsply Sirona, Ballaigues, Switzerland) according to the manufacturer’s instructions. Shaping files Nos. S1 and S2 were used in a circumferential, brushing motion until WL, then finishing files Nos. F1, F2 and/or F3 were used with a pecking motion at 250 rpm and 3 Ncm, using an X-Smart motor® (Dentsply Sirona, Ballaigues, Switzerland). The permeability file No. 10 was used at each file step. After each file was applied, the canals were irrigated with 2% NaOCl. To remove the smear layer, a final irrigation with 1 mL of 17% EDTA (Ultradent, Koln, Germany) was performed and left to act for 1 minute. It was then washed with 2 mL of 2% NaOCl. The canals were dried with paper points F2 or F3 (Dentsply Sirona, Ballaigues, Switzerland) according to the apical diameter.

For the obturation, the teeth were randomly separated into 4 groups. Top-Seal® sealer (Dentsply Sirona, Ballaigues, Switzerland) was used.

Group 1: Obturation with Thermafil. After checking the apical fit with the tester 25 or 30, 20 teeth were obturated with TF F2 or F3 after heating in the Thermaprep Plus oven® according to the manufacturer’s instructions. The sealer was inserted into the canals with paper points and then TF was placed at LT. Once the gutta-percha had cooled, a round bur was used to cut the carrier.

Group 2: Obturation with Guttacore. The apical fit was checked with a tester No. 25 or 30. The sealer was applied with a paper tip. The teeth were obturated with GuttaCore pink® No. 25 or 30, heated in the Thermaprep Plus oven® according to the manufacturer’s instructions.

Group 3: Obturation with continuous wave vertical condensation. The apical fit was checked with Protaper gutta-percha cones® Nos. F2 or F3. The sealing cement with the master gutta-percha was inserted using pumping movements. The gutta-percha was cut to 0.5 mm less than the WL. The obturation was made using the BeeFill 2-in-1® (VDW, München, Germany). The temperature was set at 200°C and the hot plugger was inserted 5 mm below the WL for obturation of the apical third. After removal, the 1-2 plugger (Dentsply Sirona Ballaigues, Switzerland) was used to condense the apical 5 mm. The middle and coronal third of the canal was obturated in several increments, using BeeFill Backfill® at a temperature of 180°C and the gutta-percha was condensed with the plugger after each increment.

Group 4: Obturation with lateral condensation technique. A master gutta-percha was used with a 2% taper and a diameter of 30 or 35, depending on the apical fit to WL. The sealer with the master gutta-percha was inserted using pumping movements. For lateral condensation, a digital spacer B and accessory gutta-perchas of 2% and diameter 20 were used.

All the teeth were stored for 7 days at 100% humidity before sample preparation.

-Preparation of samples for analysis:

The teeth were cut transversely at 5 mm and 7 mm from the apical foramen, using a low-speed handpiece with a 0.3 mm diamond disc (Komet, Lemgo, Germany) with continuous irrigation. The cross-sections were inserted in silicone putty to facilitate handling and observation. The surfaces were dried before being observed under a digital microscope Leica DMS 1000® (Leica; Wetzlar, Germany) at X6 magnification.

-Analysis of samples:

The image analysis was performed using Leica Suite® for Windows (Leica; Wetzlar, Germany). Firstly, the total area of the canal was measured, the areas occupied by gutta-percha and void areas were calculated and finally, the areas occupied by sealer.

The areas were then normalised by calculating the percentages of cement, gutta-percha and gap for each technique used, analysed at 5 mm and 7 mm.

A descriptive analysis of the percentages obtained was made using the median. To compare results in the same group at 5 mm and 7 mm, a Student’s t-test was used with a P-value <0.05, analysing the medians. To compare the different groups at 5 mm and 7 mm, the modified ANOVA test (Welch test) was performed, analysing in this case the mean (%) and standard deviation, always with a *P*<0.05 (12). In addition, the Krushal-Wallis test, with the Bonferroni correction method (*P*<0.05) was used to assess statistically significant differences between the different groups.

**Figure 1:**
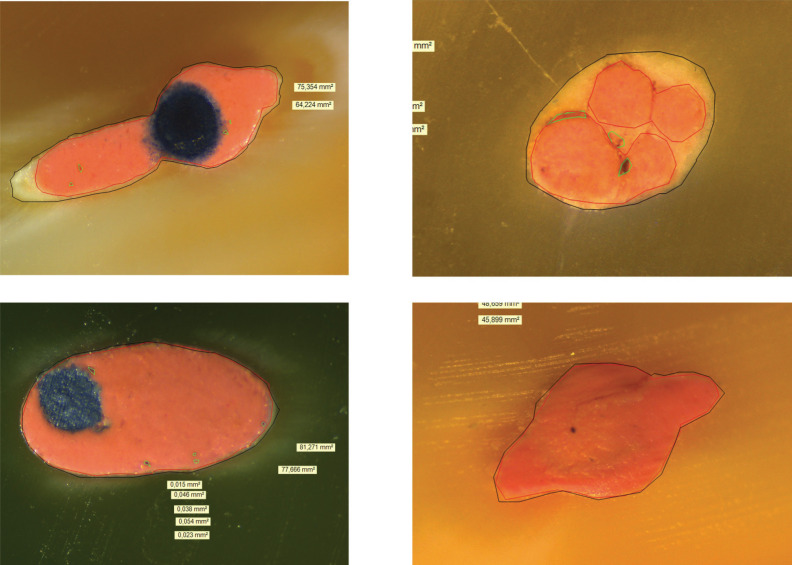
Samples at 7 mm. In black total canal area; in red gutta-percha area; in green voids. A, group 1 Thermafil; B, group 4 Lateral condensation; C, group 2 Guttacore; D group 3 continuous wave vertical condensation.

The entire statistical study was carried out with the same programme: EZR (13), (Fig. 1).

## Results

T Table 1 shows the medians of each group and the differences of each group according to the distance to the apex.

**Table 1:**
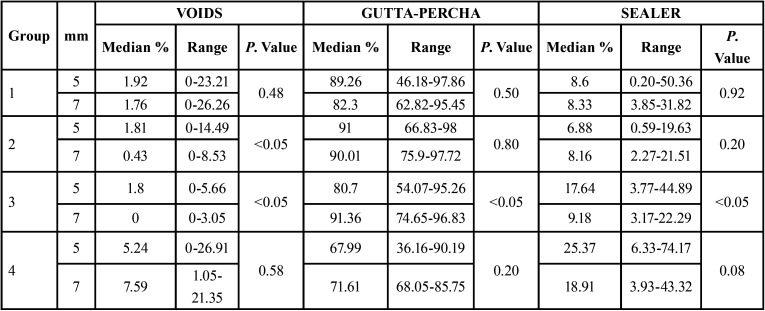
Student’s t-test. Comparison of the 4 groups at 5 mm and 7 mm, analysing the percentage of voids, gutta-percha and sealer.

In group 2 (GC), statistically significant differences were obtained in the percentage of voids (*P*<0.05) according to the distance from the apex, with a higher percentage of voids at 5 mm (1.81%).

In group 3 (VC), statistically significant differences were obtained in the percentage of voids, gutta-percha and sealer, both at 5 mm and 7 mm, with a higher amount of gutta-percha and very few voids and sealer at 7 mm.

The highest percentage of gutta-percha filled area median was found in group 3 (VC) at 7 mm, with 91.36%.

These results tend to show that, in thermoplastic techniques, there are more voids at 5 mm, while in LC there are more voids at 7 mm.

 Table 2 shows a comparison of the mean and standard deviation of the different groups at 5 mm and 7 mm. Group 3 (VC) at 7 mm shows statistically significant results in terms of fewer voids (0.33%) compared to the other groups. The LC technique obtained the lowest percentage of gutta-percha filled area at 5 mm and 7 mm. These results were statistically significant at 5 mm (66.56%) compared to the thermoplastic techniques. The highest amount of sealer was observed in group 4 (LC), with statistically significant differences with all groups at 7 mm and with groups 1 (TF) and 2 (GC) at 5 mm. Group 2 (GC) at 5 mm corresponds to the lowest amount of sealer with respect to the rest of the groups (7.82%), showing statistically significant differences with groups 3 (VC) and 4 (LC).

**Table 2:**
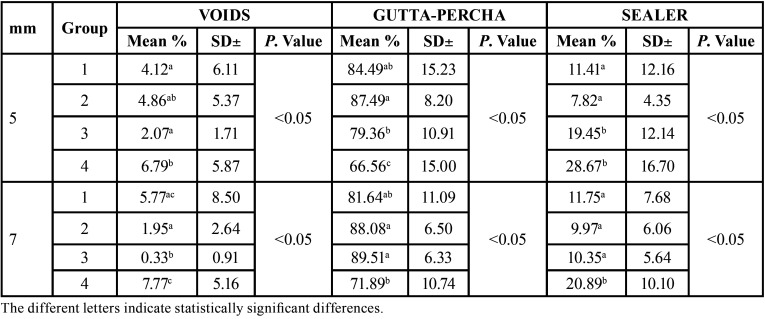
Krushal-Wallis. Comparison of the 4 groups with each other at 5 mm and 7 mm, analysing the mean, standard deviation (SD±) and *P* value.

## Discussion

The aim of this study was to quantify the gutta-percha-filled area, the sealer-filled area and the voids area in oval-shaped canals treated by thermoplastic obturation techniques compared to the LC technique. In the study, samples were examined under a Leica DMS 1000 digital microscope® (Leica; Wetzlar, Germany) and measurements were taken by digital image analysis and processing. This analysis allows for an accurate assessment of the observed root canal surfaces (14).

It was demonstrated that none of the obturation techniques used in this study achieved a gap-free root obturation, confirming previous studies (11,15). The incidence of voids in root canal fillings can be affected by many factors; the anatomical configuration, the quality of the canal preparation, the experience of the practitioner and the evaluation technique used. As noted by Keleş *et al*., resin-based sealers undergo polymerisation shrinkage, which could lead to the formation of voids (11). Therefore, the higher sealer thickness observed in the LC group would explain the higher number of voids compared to the thermoplastic techniques.

The analysis of the percentages of voids and sealer in the canals was associated with large standard deviation values, indicating a lower sealer homogeneity and consequently, a higher number of voids (11). These results are in agreement with this study, where a high standard deviation was observed for the percentage of sealer in all groups studied, except in group 2 (GC) at 5 mm. This implies a less homogeneous obturation, which may be related to the anatomy of the oval-shaped canals and the difficulty in accessing all their surfaces for preparation and subsequent obturation. Moeller *et al*. found a high correlation between microscopic examination, involving the sectioning of samples, and μCT examination of root canal obturations. Furthermore, it was found that in the μCT sections, the obturation material could be clearly distinguished from the root canal walls. The μCT provided the ability to investigate the filling quality using a detailed scale without destroying the object. The μCT images revealed several inadequately prepared and inadequately obturated areas. In fact, all the root canals had areas without root canal obturation material (15).

In the oval-shaped canals, Wu *et al*. showed that large areas of the canal wall were left uninstrumented. Owing to the irregular shape of these canals, it may be difficult to place accessory cones when using LC, so this technique may lead to more cement and gaps and consequently increase bacterial leakage (16).

Based on the premise that the best long-term seal is achieved with a minimum thickness of the sealer, a high percentage of the sealer indicates defects in the obturation. This concept stems from the fact that sealers tend to undergo setting shrinkage and dissolve over time, which may make way for reinfection of the canal area (17).

De-Deus *et al*. examined the percentages of gutta-percha area in oval-shaped canals in 50 mandibular incisors. The teeth were randomly assigned to three experimental groups including LC and thermoplastic techniques. The area occupied by gutta-percha was found to be significantly larger for all thermoplastic techniques with respect to the LC group (4). Similarly, in this study, statistically significantly higher percentages of gutta-percha filled area were found in teeth obturated with thermoplastic techniques compared to the LC technique ( Table 2).

It has been reported in one article that the Thermafil obturation technique had the lowest percentage of sealer and the highest volume of obturation material in the middle and coronal third of oval-shaped canals compared to cold obturation techniques (single cone and lateral condensation); however, in the apical third the obturation material was observed to be well adapted to the canal wall for all techniques. The TF system allowed gutta-percha to flow into the uninstrumented canal spaces, providing a better result in terms of adapting to the canal shape (17). In this study, similar results were obtained in the middle and coronal thirds in terms of the low amount of sealer and the high amount of gutta-percha in the TF group. The quality of the obturation of the samples obtained in this investigation was not analysed in the apical third because teeth usually have a rounded section at that level. These results are consistent with those of the study by Mancino *et al*., in which, at the level of the middle third, the GuttaCore and Thermafil groups had significantly more gutta-percha and less sealer compared to the continuous wave and single cone condensation group (18).

In the TF group, a relatively high standard deviation was found (±15.23) (Table 2), indicating high variability in the amount of gutta-percha in this group. These findings contradict those reported by De-Deus *et al*., who reported a very low standard deviation, giving this obturation technique greater homogeneity when filling round canals (19). Previous studies reported that the TF technique allowed the thermoplastic gutta-percha to flow better in the lateral canals, generating fewer voids and adapting to the root surface (20,21). Despite the favourable results obtained with TF, the complex anatomy of the oval-shaped canals did not allow for complete obturation without voids, as has been observed in this and other studies (22,7). However, a greater obturation capacity was observed in the TF group with respect to WL, with a significantly higher percentage of voids in the group obturated with the lateral condensation technique at 5 mm from the apex. This data does not coincide with the study by Uzunuqlu *et al*., in which the percentage of gaps in the lateral condensation technique was compared with the Herofill system, and no significant differences were found between the two techniques (23). These variations in results may be due to the different methodology used, such as the instrumentation system and the absence of sealer in the obturation.

A recent study compared the amount of voids present in both LC and GC techniques using the μCT and cross-sections. The results obtained showed statistically significant differences: both non-destructive and destructive observation methods identified significantly lower percentages of voids in the canals obturated with GC (7). Very similar results have been observed in this study. The percentage of voids obtained at both 5 mm and 7 mm in the GC group were significantly lower compared to the LC group, with values of 1.81% and 0.43% respectively, and the percentage of gutta-percha obtained was higher than 90% ( Table 1).

Among the most significant results of this study (TF, GC, VC and LC), the vertical condensation technique (VC) stood out as having the lowest percentage of voids at both 5 mm and 7 mm, the difference being statistically significant at 7 mm (0.33%). When comparing the values of voids, sealer and gutta-percha at 5 mm and 7 mm, statistically significant differences were found, with a higher percentage of gutta-percha area and a lower amount of voids and sealer at 7 mm ( Table 1). Therefore, it can be said that the obturation of the oval-shaped canals was better at 7 mm.

In this study, the samples were cut and this could be a weak point of the method used. During the sectioning process of the samples, the gutta-percha can be dragged in the contact between the cutting object and the obturated tooth, modifying the original obturation of the canal. Micro-CT is characterised by good reproducibility and accuracy for analysing root canal obturations, as the non-destructive technique provides a clear image of the samples and produces a three-dimensional view of the canal. However, axial sections obtained with micro-CT appear to have insufficient resolution to find the origin of the gap in the canal obturation when compared to microscopy studies (7). The promising development of computerised nano-tomography for the study of materials and the quantitative analysis of the quality of root canal obturations may be an interesting avenue of research to pursue (24).

## Conclusions

All techniques left areas of the canal unfilled. However, the thermoplastic techniques obtained significantly lower percentages of voids area than those obtained with the lateral condensation technique. The vertical condensation technique was the thermoplastic obturation technique with the lowest percentage of voids area with statistically significant results in the coronal third.

Based carried obturation techniques (Thermafil®, GuttaCore®) and the continuous wave vertical condensation technique achieved significantly higher percentages of gutta-percha area than the lateral condensation technique. With regard to the area occupied by sealer, both at 5 mm and 7 mm the percentage was higher for the lateral condensation technique compared to the three thermoplastic techniques.

The thermoplastic techniques can produce a better adaptation of gutta-percha in oval-shaped canals, leaving fewer empty spaces and a smaller amount of sealer in the middle and coronal thirds.

## References

[1] Mohammadi Z, Shalavi S, Jafarzadeh H (2015). The oval-shaped root canal: a clinical review. S Afr Dent J.

[2] Wu MK, R' oris A, Barkis D, Wesselink PR (2000). Prevalence and extent of long oval-shaped canals in the apical third. Oral Surg Oral Med Oral Pathol Oral Radiol Endod.

[3] Guimaraes LS, Gomes CC, Marceliano-Alves MF, Cunha RS, Provenzano JC, Siqueira JF (2017). Preparation of Oval-shaped Canals with TRUShape and Reciproc Systems: A Micro-Computed Tomography Study Using Contralateral Premolars. J Endod 2017.

[4] De-Deus G, Reis C, Beznos D, de Abranches AM, Coutinho-Filho T, Paciornik S (2008). Limited ability of three commonly used thermoplasticized gutta-percha techniques in filling oval-shaped canals. J Endod.

[5] De-Deus G, Brandao MC, Fidel RA, Fidel SR (2007). The sealing ability of GuttaFlow in oval-shaped canals: an ex vivo study using a polymicrobial leakage model. Int Endod J.

[6] Wu MK, Ozok AR, Wesselink PR (2000). Sealer distribution in root canals obturated by three techniques. Int Endod J.

[7] Li GH, Niu LN, Zhang W, Olsen M, De-Deus G, Eid AA (2014). Ability of new obturation materials to improve the seal of the root canal system: a review. Acta Biomater.

[8] Van der Sluis LW, Wu MK, Wesselink PR (2005). An evaluation of the quality of root fillings in mandibular canines using different methodologies. J Dent.

[9] De-Deus G, Murad C, Paciornik S, Reis CM, Coutinho-Filho T (2008). The effect of the canal-filled area on the bacterial leakage of oval-shaped canals. Int Endod J.

[10] Wu MK, Wesselink PR (2001). A primary observation on the preparation and obturation of oval-shaped canals. Int Endod J.

[11] Keles A, Alcin H, Kamalak A, Versiani MA (2014). Micro-CT evaluation of root filling quality in oval-shaped canals. Int Endod J.

[12] Kim HY (2014). Analysis of variance (ANOVA) comparing means of more than two groups. Resto Dent Endod.

[13] Kanda Y (2013). Investigation of the freely available easy-to-use software 'EZR' for medical statistics. Bone Marrow Transplant.

[14] De-Deus G, Maniglia-Ferreira CM, Gurgel-Filho ED, Parciornik S, Machado AC, Coutinho-Filho T (2007). Comparison of the percentage of gutta-percha-filled area obtained by Thermafil and System B. Aust Endod J.

[15] Moeller L, Wenzel A, Weggr-Larsen AM, Ding M, Kirkevang LL (2013). Quality of root fillings performed with two root filling techniques. An in vitro study using micro-CT. Act Odontol Scand.

[16] Wu MK, Van der Sluis LW, Wesselink PR (2003). The capability of two hand instrumentation techniques to remove the inner layer of dentine in oval-shaped canals. Int Endod J.

[17] Ozawa T, Taha N, Messer HH (2009). A comparison of techniques for obturating oval-shaped root canals. Dent Mat J.

[18] Mancino D, Kharouf N, Cabiddu M, Buekiet F, Haïkel Y (2021). Microscopic and chemical evaluation of the filling quality of five obturation techniques in oval-shaped root canals. Clinical Oral Investigation.

[19] De-Deus G, Gurgel-Filho ED, Magalhaes KM, Coutinho-Filho T (2006). A laboratory analysis of gutta-percha-filled area obtained using Thermafil, System B and lateral condensation. Int Endod J.

[20] Wolcott J, Himel VT, Powell W, Penney J (1997). Effect of two obturation techniques on the filling of lateral canals and the main canal. J Endod.

[21] Clinton K, Van Himel T (2001). Comparison of a warm gutta-percha obturation technique and lateral condensation. J Endod.

[22] Faus-Llácer V, Collado-Castellanos N, Alegre-Domingo T, Dolz-Solsona M, Faus-Matoses V (2015). Measurement of the percentage of root filling in oval-shaped canals obturated with Thermafil Obturators and BeeFill 2in1: In vitro study. J Clin Exp Dent.

[23] Uzunuglu E, Ilgin C, Yuruker S, Görduysus M (2016). Radiological, Stereological, and Microscopic Evaluation of the Quality of Canal Fillings in Oval-Shaped Root Canals Prepared With Self-Adjusting File. Scaning.

[24] Huang Y, Celikten B, de Faria Vasconcelos K, Ferreira Pinheiro Nicolielo L, Lippiatt N, Buyuksungur A (2017). Micro-CT and nano-CT analysis of filling quality of three different endodontic sealers. Dentomaxillofac Radiol.

